# Treatment outcome and some affecting factors of congenital adrenalhyperplasia

**DOI:** 10.1186/1687-9856-2013-S1-P120

**Published:** 2013-10-03

**Authors:** Nguyen Phu Dat, Nguyen Thuy Giang, Vu Chi Dung, Bui Phuong Thao, Nguyen Ngoc Khanh, Can Thi Bich Ngoc, Nguyen Thi Hoan

**Affiliations:** 1 Hanoi Medical University, Hanoi, Vietnam; 2 National Hospital of Pediatrics, Hanoi, Vietnam

**Keywords:** Treatment of congenital adrenal hyperplasia

## 

Congenital adrenal hyperplasia (CAH) is a common hereditary disease in the National hospital of pediatrics (NHP). CAH is treated with hormone replacement therapy for life. It is necessary to evaluate treatment outcome in order to have monitoring method and further treatment plan. Thus we study the treatment results and factors affecting treatment outcome of children with CAH being treated in the NHP. This is descriptive study. All patients diagnosed CAH being treated and monitored for more than one year at the NHP from July 1990 to July 2010. In our patient group, good outcome: 80/124 (64.52%), not good outcome: 44/124 (35.48%). Treatment outcome of patients diagnosed at the age of less than 1 year was 13.7 times better than patients diagnosed at the age of more than 1 year. Treatment outcomes were not different between males and females. Treatment outcome of a salt wasting form was 11.26 times better than that of a simple virilisation form. The group receiving a full dose had treatment results in 66.7%. A good compliance group had better treatment outcome by 8.56 times compared to that of a non-compliance group. These results of treatment depend on the age of diagnosis, using appropriate doses of hydrocortisone and treatment compliance.

